# Dendritic Cell Activation in Leprosy Using CD1a and Factor XIIIa Markers

**DOI:** 10.7759/cureus.37968

**Published:** 2023-04-22

**Authors:** Kavithamani K, Sowmya S, Shanmugasamy K

**Affiliations:** 1 Department of Pathology, Mahatma Gandhi Medical College and Research Institute, Sri Balaji Vidyapeeth (SBV) University, Pondicherry, IND

**Keywords:** bacillary index, factor xiiia, cd1a, dendritic cells, leprosy

## Abstract

Background: Leprosy is manifested in varied forms based on the immune status of the patient giving rise to the polar and borderline spectrum of tuberculoid (TT) and lepromatous leprosy (LL). The present study was conducted to assess the macrophage activation in the spectrum of leprosy using CD1a and Factor XIIIa immunohistochemical markers and to correlate the macrophage expression with the morphological spectrum and bacillary index.

Methodology: The present study was an observational study.

Results: The present study consisted of 40 biopsy-proven leprosy cases, in which a majority were males, and the most common age group was 20-40 years. The most common type encountered was borderline tuberculoid (BT) leprosy. Expression of epidermal dendritic cells and intensity of staining by CD1a was higher in TT (seven of 10 cases (70%)) when compared to LL (one of three cases (33%)). Similarly, Factor XIIIa showed higher expression of dermal dendritic cells in 90% of TT when compared to LL which was seen in 66%.

Conclusion: The increased number and strong intensity of dendritic cells in the tuberculoid spectrum may indirectly indicate macrophage activation and possibly account for the low bacillary index.

## Introduction

Leprosy or Hansen’s disease is a chronic granulomatous disease caused by obligate intracellular gram-positive bacillus *Mycobacterium leprae*. It affects mainly peripheral nerves and skin, and it can also involve the eyes, mucous membranes, bones, and testes [1]. The presence of bacilli in the skin leads to dermatological manifestations, while nerve involvement causes axonal dysfunction and demyelination thereby leading to sensory loss and its consequences such as disability and deformity. Macrophages play a key role in the pathogenesis of leprosy. In the skin, macrophages are known as dendritic cells (DCs) which are represented in the epidermis as Langerhans cells (LC) and in the dermis as dermal DCs (DD). These LC and DD can be identified by the expression of CD1a and Factor XIIIa, respectively [2]. DCs play an important role in the innate and adaptive immunity of hosts to mycobacterial infection. DCs are derived from blood monocytes [3]. CD1 is a transmembrane glycoprotein that belongs to β-microglobulin family. CD1 is structurally identical to antigen-presenting molecules of major histocompatibility complex class 1. CD1a is a marker for DCs in the epidermis. DD can be expressed by using coagulation FXIIIa (a transglutaminase) in their cytoplasm. These cells have a dendritic morphology and are seen numerously in the papillary dermis, although they are also found in the reticular and deep dermis and around blood vessels [2]. In this background the assessment of immune response is essential to analyze the course of the disease and also the management. The DC/macrophage activity is one of the indicators of immune response. The main aim of the present study is to investigate the possible role of skin DCs (macrophages) in the pathogenesis of leprosy in tissue samples obtained from patients with leprosy using immunohistochemistry.

## Materials and methods

The present study was an observational study, conducted in the Department of Pathology in a tertiary care center, Puducherry, after obtaining institutional ethical committee approval. Punch skin biopsy specimens sent from the Department of Dermatology Venereology Leprology, which were diagnosed as leprosy on histopathological examination were included in the study. The study was performed on samples received from January 2015 to May 2020. A sample size of 40 cases was taken by the convenient sampling method. The morphology of the Hematoxylin and Eosin stain was analyzed to determine the spectrum of leprosy. Fite Faraco stain was performed for calculating the bacillary index (BI) and IHC markers CD1a and Factor XIIIa were used for detecting epidermal and DD. BI was calculated based on Ridley logarithmic scale [4] as follows:

• BI 0 = No bacilli observed

• BI 1 = 1 to 10 bacilli in 10 to 100 high-power fields (hpf, oil immersion)

• BI 2 = 1 to 10 bacilli in 1 to 10 hpf

• BI 3 = 1 to 10 bacilli/hpf

• BI 4 = 10 to 100 bacilli/hpf

• BI 5 = 100 to 1,000 bacilli/hpf

• BI 6 = 1,000 bacilli/hpf (Clumps of bacilli are seen referred to as “Globi”)

Immunostaining by CD1a and Factor XIIIa was evaluated by counting the number of positive cells in the epidermis and dermis with their intensity of staining. A number of cells were expressed in percentage (%) and intensity was expressed as weak, intermediate, and strong by visual analog scale. Epidermal DCs with CD1a immunostaining were counted separately in the stratum basalis and spinosum. Data were entered in Microsoft Excel and analyzed using SSPS version 22 software. A descriptive statistical analysis was done. The results were expressed in frequencies and percentages. The chi-square test was used for categorical variables, wherever required. Results with p-value < 0.05 were considered significant.

## Results

The study was conducted on 40 cases with histopathologically proven diagnoses of leprosy at the Department of Pathology, MGMCRI. Among the 40 cases, 29 (72%) were in the tuberculoid spectrum, five (12.5%) were in the lepromatous spectrum, and one (2%) was indeterminate leprosy. Leprosy in the reaction was seen in five cases (12.5%) with type 1 upgrading reaction in four and erythema nodosum leprosum in one case. In this study, majority of cases were males (57%) and the most common age group was 20-40 years (50%).

BI-0 was seen in all cases (100%) of TT, borderline tuberculoid (BT), and IL. BI - 1+ in one case (20%) of leprosy with reaction; BI - 3+ in one case (50%) of BL; BI - 5+ in one case (50%) of BL and one case (20%) of leprosy in reaction; BI- 6+ seen in all cases (100%) of LL. BI- 2+ and 4+ were not seen in any of the cases in our study.

**Figure 1:**
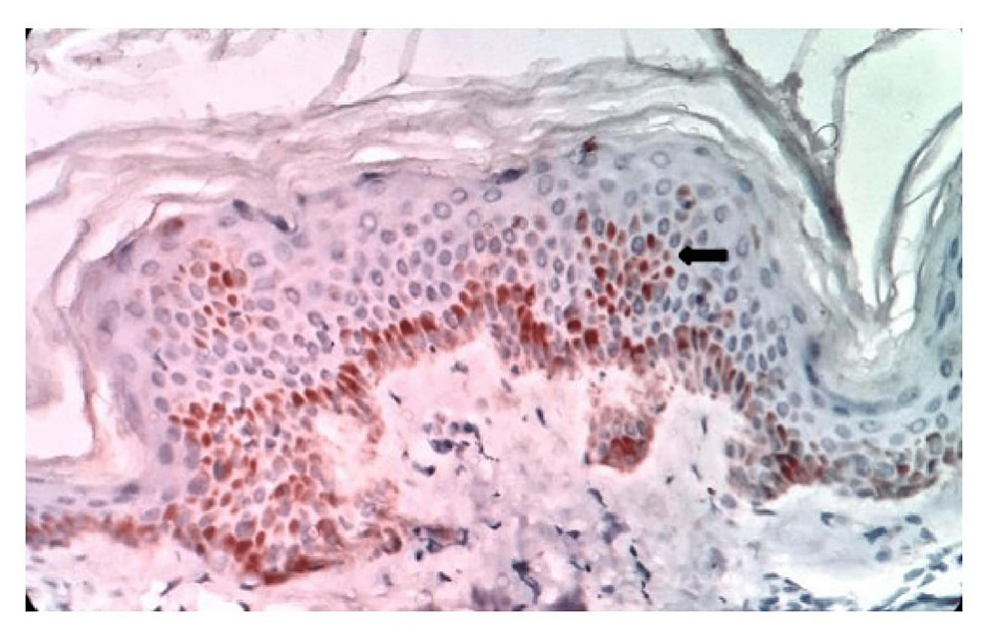
Photomicrograph of CD1a immunostaining in TT for epidermal dendritic cells in the spinous layer and keratinocytes in the basal layer, 40x. TT - Tuberculoid leprosy

**Figure 2:**
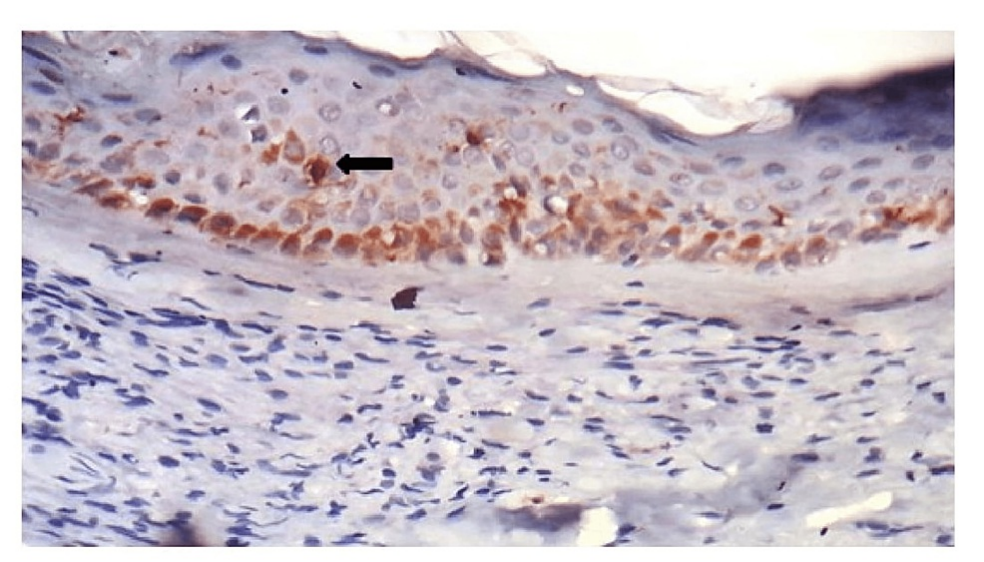
Photomicrograph of CD1a immunostaining in LL for epidermal dendritic cells in the spinous layer of LL with basal keratinocyte staining, 40x. LL - Lepromatous leprosy

CD1a staining in the spinous layer of the epidermis was seen in seven of 10 cases (70%) in TT (Figure 1), nine of 19 cases (47%) in BT, one of two cases (50%) in BL, one of three cases (34%) in LL (Figure 2) and one of five cases (20%) in lepra reaction. The highest number (11%-20%) of epidermal DCs stained by CD1a was observed in two cases of TT and one case of BT leprosy (Table 1). The variable-staining intensity with CD1a was observed in the basal layer of the epidermis in all the cases. Dermal dendrocytes showed strong intensity positive with CD1a in two cases (20%) of TT and nine cases (47%) of BT (Table 2).

**Table 1:** CD1a immunostaining pattern, intensity, and % of dendritic cells stained in the spinous layer of epidermis.

Spectrum		TT	BT	BL	LL		IL		Reaction		P-value
Total no. of cases		10	19	02	03		01		05		0.861 0.867
CD1a Staining pattern	1) Epidermis with only basal layer staining	3 (30%)	10 (53%)	1 (50%)	2 (66%)		1 (100%)		4 (80%)		
	2) Epidermis showing basal and spinous staining	7 (70%)	9 (47%)	1 (50%)	1 (34%)		0%		1 (20%)		
Intensity											
	1) Weak	2 (20%)	5 (24%)	0%	0%		0%		1 (20%)		
	2) Intermediate	5 (50%)	12 (66%)	2 (100%)	2 (66%)		1 (100%)		3 (60%)		
	3) Strong	3 (30%)	2 (10%)	0%	1 (34%)		0%		1 (20%)		
% of cells stained in spinous layer											
	Negative	3 (30%)	10 (53%)	1 (50%)		2 (66%)		1 (100%)		4 (80%)	
	1-10%	5 (50%)	8 (42%)	1 (50%)		1 (33%)		0%		1 (20%)	
	11-20%	2 (20%)	1 (5%)	0%		0%		0%		0%	

**Table 2:** CD1a pattern and intensity of immunostaining of dermal dendritic cells throughout the spectrum of leprosy.

Spectrum		TT	BT	BL	LL	IL	Reaction	P-value
Total cases		10	19	02	03	01	05	0.489
CD1a Staining pattern	Scattered staining in papillary dermis	3 (30%)	4 (20%)	0%	0%	0%	2 (40%)	
	Scattered staining in deep dermis	0%	5 (26%)	0%	0%	0%	0%	
	Negative staining in the dermis	7 (70%)	10 (54%)	2 (100%)	3 (100%)	1 (100%)	3 (60%)	
Intensity								0.907
	Strong	2 (20%)	2 (10%)	0%	0%	0%	1 (20%)	
	Intermediate	0%	3 (16%)	0%	0%	0%	1 (20%)	
	Weak	1 (10%)	4 (20%)	0%	0%	0%	0%	

**Figure 3:**
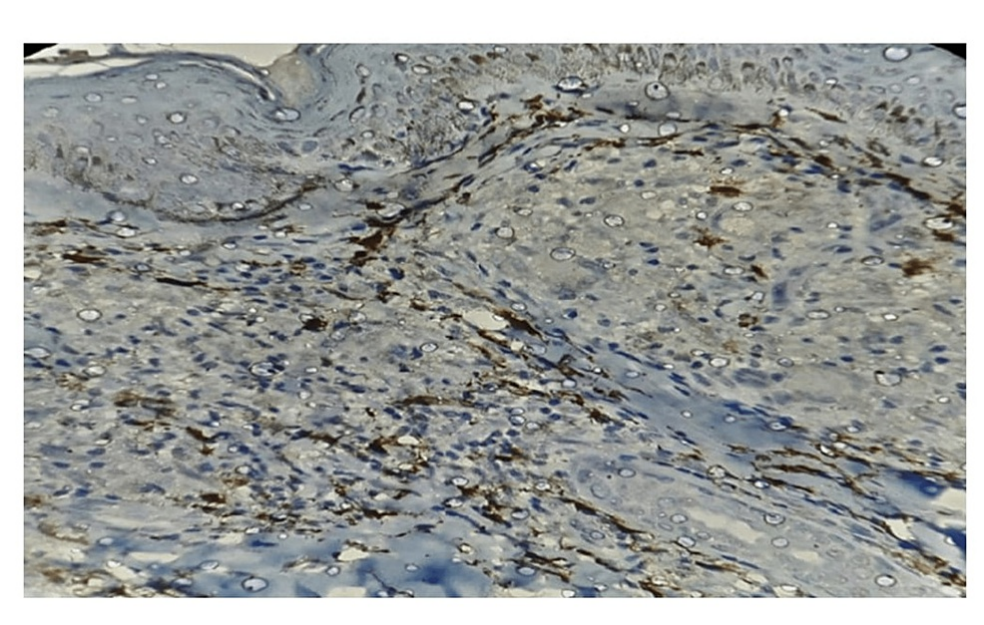
Photomicrograph showing Factor XIIIa immunostaining in TT of dermal dendritic cells around the granuloma in papillary dermis, 40x. TT - Tuberculoid leprosy

**Figure 4:**
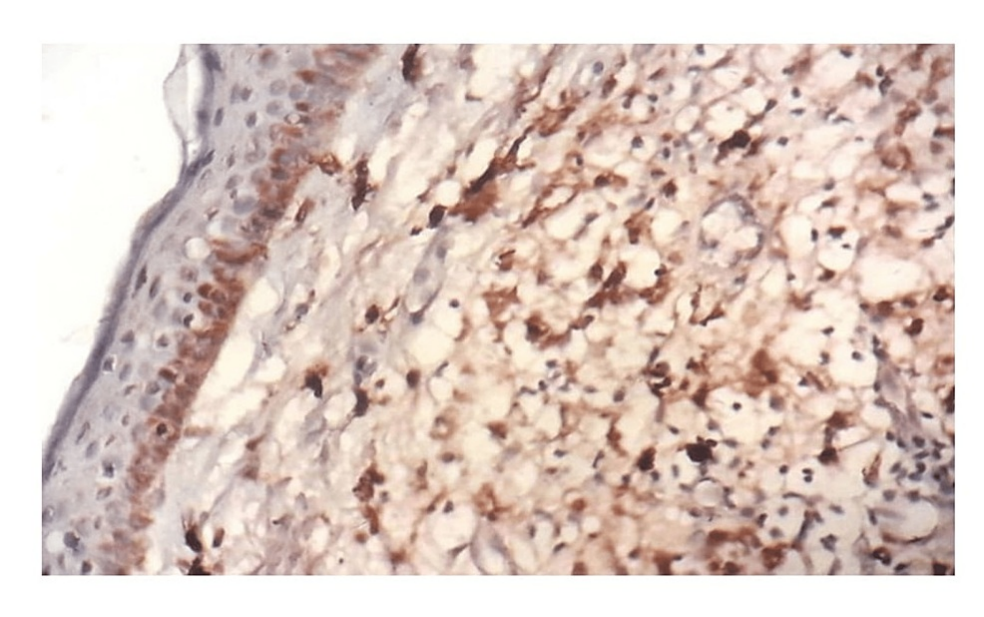
: Photomicrograph showing scattered strong intensity Factor XIIIa immunostaining of dermal dendritic cells throughout the dermis in LL, 40x. LL - Lepromatous leprosy

Factor XIIIa showed perigranulomatous staining pattern (Figure 3) both in papillary and deep dermis was seen in four cases (21%) of BT, two cases (20%) of TT and two cases (40%) in reaction. Scattered staining in the dermis in 15 cases of tuberculoid spectrum and two cases of lepromatous spectrum (Figure 4). One case each in TT and LL showed negative staining in the dermis. More than 80% of Factor XIIIa positive DD was observed in four cases (40%) of TT, one case (5%) of BT and one case (20%) in upgrading reaction (Table 3).

**Table 3:** Factor XIIIa immunostaining pattern, intensity, and % of dermal dendritic cells stained throughout the spectrum of leprosy.

Spectrum		TT	BT	BL	LL	IL	Reaction	P-value	
Total cases	N	10	19	02	03	01	05	0.598	
FACTOR XIIIA Staining pattern									
	1.Scattered staining in dermis	6 (60%)	9 (47%)	1 (50%)	1 (34%)	1 (100%)	2 (40%)		
	2. Papillary dermis showing staining around the granulomas.	1 (10%)	6 (32%)	1 (50%)	1 (33%)	0%	1 (20%)		
	3. In both papillary & deep dermis staining is seen around the granulomas	2 (20%)	4 (21%)	0%	0%	0%	2 (40%)		
	4. Negative staining	1 (10%)	0%	0%	1 (33%)	0%	0%		
Intensity								0.196	
	1.Weak	0%	0%	0%	0%	0%	1 (20%)		
	2.Intermediate	2 (20%)	5 (26%)	0%	0%	1 (100%)	2 (40%)		
	3.Strong	7 (70%)	14 (74%)	2 (100%)	2 (66%)	0%	2 (40%)		
% of cells stained									
	Negative	1 (10%)	0%	0%	1 (33%)	0%	0%		
	20-50%	1 (10%)	11 (57%)	0%	0%	1 (100%)	3 (60%)		
	51-80%	4 (40%)	7 (36%)	2 (100%)	2 (66%)	0%	1 (20%)		
	>80%	4 (40%)	1 (5%)	0%	0%	0%	1 (20%)		

Factor XIIIa immunostain was positive in epidermal basal keratinocytes with variable staining intensity in all cases except for one case in reaction. CD1a staining in both basal and spinous layer with variable intensity with BI 0 was seen in 17 cases (54%), one case each of BI 3 and BI 6 showed intermediate staining. CD1a in dermis with BI 0 showed scattered staining with variable intensity in papillary dermis in eight cases (27%), scattered staining with variable intensity in deep dermis was seen in five cases (15%). All the cases with BI more than zero were negative for CD1a in the dermis. The expression of epidermal DCs by Factor XIIIa showed variable presentation in relation to BI.

## Discussion

The present study was performed on 40 histopathologically proved cases of leprosy and the morphological patterns were analyzed and correlated with the immunohistochemical profile of DCs stained by CD1a and Factor XIIIa. In this study, epidermal basal keratinocyte positivity in all the cases was seen with CD1a immunostaining throughout the spectrum. Furue et al, observed that BL-6 which is a type of CD1a antibody was strongly expressed on keratinocytes and this staining was prominent in the follicular infundibulum [5], whereas spinous layer staining indicated the presence of DCs. Spinous layer staining representing the epidermal DCs was predominantly seen in the tuberculoid spectrum in the present study (Table 1).

CD1a expression was comparatively less in lepromatous leprosy (LL) which was similar to the study conducted by Sieling et al. This weak expression of CD1a in lepromatous lesions was not because of the primary defect of the CD1 system itself, but there may be some local factors at the site of infection that may be responsible for the blockade of CD1a expression in lepromatous cells [6]. Quantitative analysis of CD1a by Hirari et al. also showed a similar expression of DCs in the epidermis [7].

Dermal staining of CD1a was not much documented in the literature. It was considered an immunostain for epidermal DCs. However, in the present study, a few cases of the tuberculoid spectrum showed scattered CD1a-positive cells with strong intensity in the papillary dermis in two cases (20%) of TT and nine cases (47%) of BT (Table 2).

**Figure 5:**
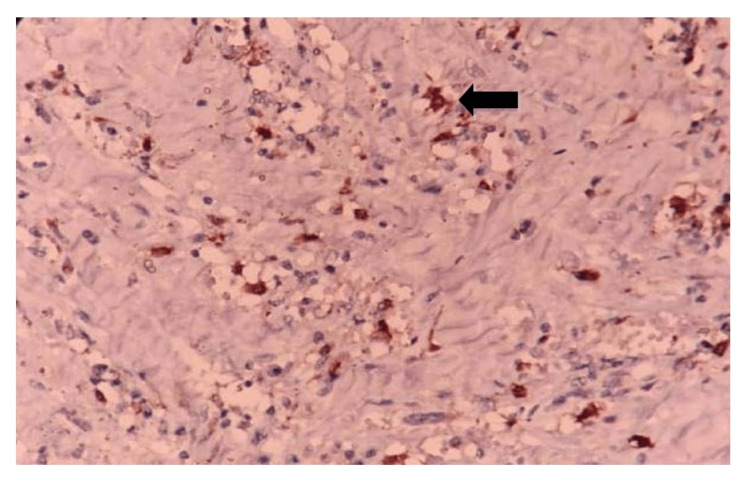
Photomicrograph showing cytoplasmic projections with Factor XIIIa immunostain in dermal dendritic cells, 40x.

Factor XIIIa in the present study showed cytoplasmic staining of DD with visible dendritic projections (Figure 5). Perigranulomatous-staining pattern both in the papillary and deep dermis was seen in four cases (21%) of BT, two cases (20%) of TT, and two cases (40%) in reaction. The present study was in concordance with a study conducted by Quaresma et al. [2] and Hirari et al [7] in which the expression of FXIIIa with more number of DD was observed in TT leprosy compared to LL.

Hirari et al. also observed that FXIIIa staining in the skin fragments of leprosy patients was mainly detected in the dermis, with a large number of positive cells in the papillary dermis, at the dermoepidermal junction and around blood vessels and granulomas. The cytoplasm of these cells had clearly visible dendritic prolongations which were elongated, spindle-shaped, or oval. This pattern of staining of factor XIIIa in the dermis was in concordance with the present study. Quantitative analysis of Hirari et al. showed that despite the larger number of FXIIIa positive DCs seen in tuberculoid form, there was no significant difference observed between the different poles of the disease [7].

The study by Miranda et al. observed that there was an increased accumulation of LC after mycobacterial stimulation. And they also noted that there was a significant number of LC in patients with a reversal reaction and erythema nodosum leprosum, similar to the findings seen in tuberculoid lesions. In the same study, they also observed that the number of LC was considerably lower in LL compared to the other groups which were concordant with the present study [8].

Factor XIIIa immunostain was positive for epidermal basal keratinocytes in all cases except for one case in reaction. Factor XIIIa staining was negative in the spinous layer of the epidermis. A study conducted by Paragh and Torocsik observed that a clone AC-1A1, which is a mouse monoclonal antibody for FXIIIa showed positive staining for keratinocytes depending on their maturation. They also observed that the same clone of Factor XIIIa can also express fibroblasts and Sebocytes [9]. This supports the keratinocytic staining by Factor XIIIa in the present study. The basal keratinocytes were stained by both CD1a and Factor XIIIa. Since the DCs were indistinguishable from keratinocytes in the basal layer, the CD1a-positive cells in the spinous layer were counted as DCs. This resulted in comparatively a lesser number of cells (maximum number of 11% to 20%) in the epidermis than in the dermis (maximum number of >80%). Further immune markers are required to differentiate DCs from the keratinocytes in the basal layer. Epidermal DCs stained with CD1a were observed in 12 cases with BI-0. DD with CD1a showed scattered staining with variable intensity in the papillary dermis in eight cases (27%), scattered staining with variable intensity in the deep dermis in five cases (15%) and showed negative staining in cases with BI more than zero. Factor XIIIa showed scattered DD with strong intensity staining in 14 cases with BI-0. The expression of epidermal DCs by Factor XIIIa showed a variable presentation in relation to BI. CD1a and Factor XIIIa correlation with BI was not much documented in the literature. Epidermal and DD showed stronger staining intensity and a higher number of expressions by both CD1a and Factor XIIIa in the tuberculoid spectrum compared to the lepromatous spectrum. Even though the number may not be sufficient to arrive at a conclusion, the findings might indicate that the increased number of DCs both in the epidermis and the dermis observed predominantly in the tuberculoid spectrum may indicate the activation of macrophage system to combat the organism. Thereby the DCs have a pivotal role in determining the paucibacillary or multibacillary nature of the disease. The intensity of the immune response gives rise to the spectrum of clinical manifestation and hence has implications in the course of management of leprosy.

Limitations of the study

Quantification of DCs was done by a visual analog scoring system and not by using a grid; hence, there may be subjective variation. An attempt to overcome this limitation was done by three persons counting and comparing the numbers independently. The number of macrophages and staining intensity were only indirect evidence of the activation of macrophages. The study was conducted only on samples with a histopathological diagnosis of leprosy and no control group was taken for comparison.

## Conclusions

BT was the most common type of leprosy. Even though statistically not significant, the tuberculoid spectrum showed more number of dermal and epidermal DCs with strong intensity when stained with CD1a and Factor XIIIa in comparison to the lepromatous spectrum. This may indicate the possible role of macrophage activation in the tuberculoid spectrum in mounting an immune response against the lepra bacilli.
